# The Modification Effect of Influenza Vaccine on Prognostic Indicators for Cardiovascular Events after Acute Coronary Syndrome: Observations from an Influenza Vaccination Trial

**DOI:** 10.1155/2016/4097471

**Published:** 2016-04-20

**Authors:** Apirak Sribhutorn, Arintaya Phrommintikul, Wanwarang Wongcharoen, Usa Chaikledkaew, Suntara Eakanunkul, Apichard Sukonthasarn

**Affiliations:** ^1^Ph.D. Program in Clinical Epidemiology, Faculty of Medicine, Chiang Mai University, Chiang Mai 50200, Thailand; ^2^Department of Pharmacy Practice, School of Pharmaceutical Sciences, University of Phayao, Phayao 56000, Thailand; ^3^Cardiology Division, Department of Internal Medicine, Faculty of Medicine, Chiang Mai University, Chiang Mai 50200, Thailand; ^4^Social and Administrative Pharmacy Excellence Research (SAPER) Unit, Department of Pharmacy, Faculty of Pharmacy, Mahidol University, Bangkok 10400, Thailand; ^5^Department of Pharmaceutical Sciences, Faculty of Pharmacy, Chiang Mai University, Chiang Mai 50200, Thailand

## Abstract

*Introduction*. The prognosis of acute coronary syndrome (ACS) patients has been improved with several treatments such as antithrombotics, beta-blockers, and angiotensin-converting enzyme inhibitors (ACEI) as well as coronary revascularization. Influenza vaccination has been shown to reduce adverse outcomes in ACS, but no information exists regarding the interaction of other treatments.* Methods*. This study included 439 ACS patients from Phrommintikul et al. A single dose of inactivated influenza vaccine was given by intramuscular injection in the vaccination group. The cardiovascular outcomes were described as major cardiovascular events (MACEs) which included mortality, hospitalization due to ACS, and hospitalization due to heart failure (HF). The stratified and multivariable Cox's regression analysis was performed.* Results*. The stratified Cox's analysis by influenza vaccination for each cardiovascular outcome and discrimination of hazard ratios showed that beta-blockers had an interaction with influenza vaccination. Moreover, the multivariable hazard ratios disclosed that influenza vaccine is associated with a significant reduction of hospitalization due to HF in patients who received beta-blockers (HR = 0.05, 95% CI = 0.004–0.71, *P* = 0.027), after being adjusted for prognostic indicators (sex, dyslipidemia, serum creatinine, and left ventricular ejection fraction).* Conclusions*. The influenza vaccine was shown to significantly modify the effect of beta-blockers in ACS patients and to reduce the hospitalization due to HF. However, further study of a larger population and benefits to HF patients should be investigated.

## 1. Introduction

Influenza vaccination in the community can significantly reduce influenza infection [1] and incidence of influenza-like illness among the elderly [2], as well as hospitalization and death due to pneumonia, influenza [3–7], or cardiovascular diseases [1, 3–8]. Furthermore, randomized controlled studies have demonstrated benefits in reducing major adverse cardiovascular events among patients with coronary artery diseases (CAD) [9–13]. For this reason, the American Heart Association and American College of Cardiology recommend influenza vaccination as a secondary prevention intervention in patients with CAD and atherosclerotic vascular diseases [14, 15] and those with ST-segment elevation myocardial infarction (STEMI) [16] and unstable angina/non-STEMI [17] as well as a plan of care for patients with chronic heart failure [18].

Nonetheless, the evidence-based recommendations and benefits of influenza vaccination have been shown in CAD; the mechanisms of its benefit have not yet been defined, as well as some queries on the vaccine immunological response in patients with various clinical characteristics, such as impaired renal function or concurrent medications [19–22]. The study of prognostic indicators and patients' clinical characteristics may describe the benefits of influenza vaccine for cardiovascular outcomes.

An annual influenza vaccination can prevent influenza virus infection and relieve the symptoms of acute infection. In fact, an annual influenza vaccination can prevent influenza infection and also decrease the results from acute infection, where it promotes inflammation and the progression of atherosclerosis and it serves as a trigger for acute myocardial infarction [23–29]. Consequently, the administration of influenza vaccine may reveal an influence on some prognostic indicators for cardiovascular outcomes, compared with patients not receiving the vaccination.

Therefore, this study aimed to explore the effects of the influenza vaccine through the prognostic indicators for each cardiovascular outcome among ACS patients.

## 2. Patients and Methods

### 2.1. Data Sources and Data Collection

This observational study was based on a prospective, randomized open with blinded endpoint study from Phrommintikul et al. [9], which enrolled 439 patients who had been admitted due to ACS and were older than 50 years old. Patients were excluded if they had hemoglobin level lower than 10 g/dL, elevated serum creatinine (SCr) level more than 2.5 mg/dL, well-established liver disease, cancer or life expectancy less than one year, and contraindications to, or previous, influenza vaccination. All patients were given standard treatment by their primary cardiologist in the tertiary care hospital of Chiang Mai University.

### 2.2. Definition

The ACS patients were classified into three groups. These included the following: (1) patients with an acute ST-segment elevation myocardial infarction (STEMI) described as a chest pain lasting longer than 20 minutes with ST-segment elevation of electrocardiograph (EKG) in two consecutive leads or more, (2) patients with chest pain lasting longer than 20 minutes, with rising of cardiac troponin or CK-MB and without ST-segment elevation EKG, defined as non-ST-segment elevation myocardial infarction (NSTEMI), and (3) patients with chest pain at rest without rising of cardiac troponin or CK-MB, diagnosed as an unstable angina (UA), whereas NSTEMI and UA were defined as non-ST-segment elevation ACS (NSTE-ACS).

The studied patients' characteristics included age, sex, concurrent comorbidities, that is, hypertension (HT); diabetes mellitus (DM); dyslipidemia; chronic obstructive pulmonary disease (COPD); smoking; prior myocardial infarction (MI); chronic kidney disease (CKD), SCr, type of ACS, revascularization procedure, left ventricular ejection fraction (LVEF), and medications.

The main cardiovascular outcomes of interest were defined as (1) major adverse cardiovascular events (MACEs), a composite of all cardiovascular events, (2) all causes of mortality, (3) hospitalization due to acute coronary syndrome (ACS), (4) hospitalization due to heart failure (HF), and (5) composite outcomes of hospitalization (ACS, HF, or stroke). These outcomes were verified by cardiologists during the follow-up of 12 months. Survival status of patients lost to follow-up was determined by telephone.

### 2.3. Data Analysis

The patients' characteristics were compared among five types of adverse cardiovascular outcomes and each outcome-free group, using Fisher's exact test, where multiple imputations were manipulated for missing data management.

Prognostic indicators for each cardiovascular outcome were stratified by influenza vaccine groups and analyzed as multivariable hazard ratio by the stratified Cox regression.

The *Z*-test was performed to demonstrate significant discrimination of hazard ratio between influenza vaccination groups [30].

Multivariable Cox's regression was conducted to present the results, subsequently adjusted for independent prognostic indicators of each cardiovascular outcome.

This study was approved by the Ethics Committee, Faculty of Medicine, Chiang Mai University.

## 3. Results

### 3.1. Patients' Characteristics

In this observational study, data of 439 ACS patients were collected. Half of the patients were older than 65 years old and 56.7% of the patients (249) were males (Table 1). HT was present among 265 (60.4%); DM, 134 (30.5%); dyslipidemia, 206 (46.9%); COPD, 13 (3.0%); and CKD, 20 (4.56%). Regarding the index ACS, STEMI and NSTE-ACS were present among 159 (36.2%) and 280 (63.8%) of the patients, respectively. The majority of STEMI patients (79.25%) received reperfusion therapy and more than a half of the NSTE-ACS patients (53.21%) received coronary revascularization. Aspirin, beta-blockers, and statin were received among 427 (97.3%), 325 (74.0%), and 293 (66.7%) patients, respectively.

### 3.2. Prognostic Indicators of Adverse Outcomes

The characteristics of ACS patients with and without MACEs were not significantly different, except for dyslipidemia, LVEF, receiving angiotensin-converting enzyme inhibitors (ACE-I) or angiotensin receptor blockers (ARB), and influenza vaccination (Table 1). Patients with MACEs had higher proportion of dyslipidemia (61.3% versus 44.6%, *P* = 0.019) but a lower proportion of receiving ACE-I/ARB (45.2% versus 60.7%, *P* = 0.026) and influenza vaccination (33.9% versus 53.1%, *P* = 0.006). The MACEs-free patients also had a great proportion of preserved LVEF (LVEF > 40%) (70.8% versus 51.6%, *P* = 0.005) (Table 1).

Regarding the causes of death, patients who survived were younger (age 65 ± 9.17 versus 73.0 ± 9.29 years, *P* = 0.0014). The other clinical characteristics did not significantly differ between two groups (Table 1).

When comparing between patients with composite outcomes of hospitalization due to ACS, HF, or stroke and those who were not hospitalized (Table 2), patients with these events had a higher proportion of dyslipidemia (63.3% versus 44.9%, *P* = 0.022) and impaired LVEF (LVEF < 40%) (49.0% versus 29.7%, *P* = 0.009). They also had low proportion of influenza vaccination (32.7% versus 52.6%, *P* = 0.010) (Table 2).

The comparison of three outcomes among those hospitalized due to ACS, HF, and event-free patients revealed significant differences in proportion of dyslipidemia (58.9%, 78.6%, and 44.8%, resp., *P* = 0.017), CKD (2.9%, 28.6%, and 3.8%, resp., *P* = 0.004), impaired LVEF (35.3%, 78.6%, and 29.9%, resp., *P* = 0.001), and influenza vaccination (32.35%, 28.57%, and 52.69%, resp., *P* = 0.020) (Table 2). Interestingly, patients hospitalized due to HF had a high proportion of dyslipidemia (78.6%, *P* = 0.017), presented CKD (28.6%, *P* = 0.004), and impaired LVEF (78.6%, *P* = 0.001) but revealed a lower proportion of receiving influenza vaccination (28.6%, *P* = 0.020).

When stratified Cox's regression analysis by influenza vaccine group was performed for each cardiovascular outcome (Table 3), the significant protective indicator was receiving ACE-I/ARB, while impaired LVEF, age above 65 years, and CKD presented poor indicators in the nonvaccination group.

The impaired LVEF variables were shown as poor prognostic indicators in both groups of patients with similar hazard ratios (Tables 3 and 4). Age above 65 years was indicated as a significant prognostic indicator for death in the nonvaccination group (HR = 10.78, 95% CI = 1.39–83.62, *P* = 0.023) but not in the vaccination group (HR = 2.28, 95% CI = 0.42–12.48, *P* = 0.341). However, the effect size of age did not significantly vary between vaccination groups (*P* = 0.252) (Table 4). Differently, the CKD variable was a promising poor prognostic indicator in both groups, (HR = 5.12, 95% CI = 1.27–20.65, *P* = 0.022) and (HR = 24.01, 95% CI = 1.34–417.20, *P* = 0.029). However, the effect size of CKD hazard ratio seemed to diverge with a wide range of confidence intervals; a significant difference was not demonstrated (*P* = 0.340) (Table 4).

Receiving beta-blockers was shown as a nonprotective indicator as well as demonstrating no prognostic value in the nonvaccination group, but it was shown as a potential protective indicator in the vaccination group (HR = 0.05, 95% CI = 0.003–0.76, *P* = 0.037) (Table 3). Moreover, the comparison of hazard ratio between vaccination groups indicated a remarkable difference (*P* = 0.03) (Table 4).

In summary, the influenza vaccination influenced the prognostic value of clinical predictors for each cardiovascular outcome when compared with nonvaccination group, except two predictors of impaired LVEF for MACEs (HR = 2.07, 95% CI = 1.12–3.82, *P* = 0.021 and HR = 2.37, 95% CI = 1.01–5.59, *P* = 0.048) and CKD for hospitalization due to HF (HR = 5.12, 95% CI = 1.27–20.65, *P* = 0.022 and HR = 24.01, 95% CI = 1.34–417.20, *P* = 0.029). However, no significant difference was observed of hazard ratios between influenza vaccination groups, but receiving beta-blockers revealed the differences (*P* = 0.030) (Table 4).

Multivariable Cox's regression (Table 5) demonstrated that influenza vaccination and beta-blockers coadministration indicated a potential protective effect (HR = 0.05, 95% CI = 0.004–0.71, *P* = 0.027) after adjusting for sex, dyslipidemia, CKD, SCr, and LVEF, but both factors were independent prognostic indicators for hospitalization due to HF.

The interaction of influenza vaccination among patients receiving beta-blockers was described by a significant reduction of the hazard ratio among patients who had vaccination. This protective interaction showed benefits of receiving influenza vaccination with beta-blocker for hospitalization due to HF among ACS patients.

## 4. Discussion

This post hoc study demonstrated that the significant prognostic indicators for cardiovascular events in patients with ACS were age, LVEF, CKD, and receiving ACE-I/ARB. Even though the hazard ratio of each individual prognostic factor may differ between the vaccination and nonvaccination groups, the difference was not significant, except for receiving beta-blockers. Receiving beta-blockers presented the prognostic indicator for the reduction of hospitalization due to HF when influenza vaccine was given.

The evidence from seasonal patterns of cardiovascular deaths was similar to patterns of influenza circulation [29]. Clinical findings among patients with influenza presented systemic effects such as myalgia, high fever, and fatigue, as well as frequent myocardial involvement [29]. The influenza virus has extensive effects on the inflammatory and coagulation pathways, leading to destabilization of vulnerable atherosclerotic plaques and coronary occlusion, which are major causes of acute MI [29]. Moreover, host response to acute infections can facilitate ACS by affecting coronary arteries and atherosclerotic lesions, such as increased sympathetic activity [28].

The upregulated sympathetic nervous system shown in heart failure [18] may reduce the influenza vaccine response [31–33] or cause persistence decline of antibody titers [32].

The sympathetic nervous system will increase proinflammatory cytokines and exacerbate influenza infection, as shown in animal models [34]. In the lung of infected animals, the anti-influenza CD8+ T cell response could be limited by sympathetic nervous system [35], while cytotoxic T lymphocytes could effectively respond to different subtypes of influenza A virus with a specific antibody response [36]. Cytotoxic T cells were described as important factors for recovering from influenza infection in humans [36].

Human T and B lymphocytes express beta-2 adrenergic receptors, where the catecholamine effect via beta-2 adrenergic receptors on cytokine regulation decreased responses to vaccines [37]. In contrast, T cell responses were enhanced by the administration of beta-2 adrenergic antagonists [35].

The study in mice showed that acute stress reduced the number of NK cells in the intraparenchymal region of the lungs and this event could be reversed by the administration of beta-adrenergic antagonists [38]. Acute stress can be hypothesized as the cause of lung lymphocyte redistribution through beta-adrenergic stimulation by elevating catecholamine level [38]. Therefore, beta-blockers could reduce the inflammatory response and the degree of lung injury. Some animal models revealed survival benefits, particularly when beta-blockers were administered before the septic insult [39].

Beta-blockers are recommended as a secondary prevention for ACS patients recovering from acute MI and without contraindication [40]. ACS was indicated as an important cause of worsening or new-onset of HF and also a common factor precipitating acute decompensated HF [18]. Consequently, prescribing beta-blockers to chronic HF patients is recommended due to their protective results [18, 41].

The decrease in heart rate, contractility, and blood pressure due to beta-blockers could inhibit the effects of circulating catecholamines and oxygen demand [42]. Beta-blockers can reduce the sympathetic tone by inhibiting an increase in catecholamine circulation [43], as a cause of proinflammatory cytokines [34, 43] and disrupt the immune response [43].

Moreover, the administration of influenza vaccine can prevent influenza infection and also reduce acute infection effects by promoting inflammation, the progression of atherosclerosis, and triggering acute MI [9–15, 17].

In this study, solely administration of beta-blockers or influenza vaccination was not shown to be the protective evidence for hospitalization due to HF among ACS patients. However, the combination of the two showed very synergistic effect during a year of follow-up time.

### 4.1. Limitation

Incomplete data was a limitation of this study. Only 2 incomplete variables were found from 20 variables. The variables of SCr and LVEF had 6.83% and 54.67% of missing values, respectively. However, multiple imputations were conducted and imputed data were categorized for appropriate data management.

## 5. Conclusion

The study showed that influenza vaccination influenced the prognostic abilities of clinical predictors for cardiovascular outcomes when compared between patients who received vaccination and the nonvaccination group. However, two predictors of impaired LVEF for MACEs and CKD for hospitalization due to HF were not affected. Moreover, different prognostic ability between influenza vaccination groups was not significantly observed, but receiving beta-blockers was acknowledged.

This study presented the strong modification effect of influenza vaccine among ACS patients who received beta-blockers to reduce hospitalization due to HF. This benefit of influenza vaccination should be noteworthily considered in clinical practice for ACS patients. However, further studies of influenza vaccine and beta-blocker synergy should be established in a larger population involving clinical trials.

Although, this study disclosed a new benefit of influenza vaccine and beta-blockers coadministration in preventing HF hospitalization, a further study involving influenza vaccine among HF patients is strongly recommended.

## Figures and Tables

**Table 1:** Patients' characteristics for MACEs and death.

Characteristics	Total (*n* = 439)		Event-free (A) (*n* = 377)		MACEs (*n* = 62)		*P* value	Survived (*n* = 421)		Death (*n* = 18)		*P* value
	*n*	%	*n*	%	*n*	%		*n*	%	*n*	%	
Age (year)												
≤65	219	49.9	194	51.5	25	40.3	0.131	216	51.3	3	16.7	0.006
>65	220	50.1	183	48.5	37	59.7		205	48.7	15	83.3	

Male	249	56.7	218	57.8	31	50.0	0.270	243	57.7	6	33.3	0.052
HT	265	60.4	222	58.9	43	69.4	0.126	252	59.9	13	72.2	0.336
DM	134	30.5	113	30.0	21	33.8	0.553	127	30.2	7	38.9	0.440
Dyslipidemia	206	46.9	168	44.6	38	61.3	0.019	197	46.8	9	50.0	0.814
COPD	13	3.0	11	2.9	2	3.2	1.000	13	3.1	0	0.0	1.000
Smoking	48	11.0	45	11.9	3	4.8	0.123	48	11.4	0	0.0	0.241
Prior MI	18	4.1	15	4.0	3	4.8	0.729	18	4.3	0	0.0	1.000
CKD	20	4.6	15	3.9	5	8.1	0.181	20	4.8	0	0.0	1.000

SCr (mg/dL)												
≤1.1	221	50.3	194	51.5	27	43.6	0.274	212	50.4	9	50.0	1.000
>1.1	218	49.7	183	48.5	35	56.5		209	49.6	9	50.0	

Type of ACS												
NSTEMI & UA	280	63.8	242	64.2	38	61.3	0.671	272	64.6	8	44.4	0.130
STEMI	159	36.2	135	35.8	24	38.7		149	35.4	10	55.6	

Reperfusion or revascularization												
No	164	37.4	141	37.4	23	37.1	1.000	158	37.3	6	33.3	0.808
Yes	275	62.6	236	62.6	39	62.9		263	62.5	12	66.7	

LVEF (%)												
>40	299	68.1	267	70.8	32	51.6	0.005	290	68.9	9	50.0	0.120
≤40	140	31.9	110	29.2	30	48.4		131	31.1	9	50.0	

Medication												
Aspirin	427	97.3	366	97.1	61	98.4	1.000	409	97.2	18	100.0	1.000
*β*-blocker	325	74.0	281	74.5	44	71.0	0.536	311	73.9	14	77.8	1.000
CCB	72	16.4	63	16.7	9	14.5	0.853	69	16.4	3	16.7	1.000
ACE-I/ARB	257	58.5	229	60.7	28	45.2	0.026	250	59.4	7	38.9	0.093
Statin	293	66.7	252	66.8	41	66.1	1.000	283	67.2	10	55.6	0.315
Influenza vaccination	221	50.3	200	53.1	21	33.9	0.006	215	51.1	6	33.3	0.156

DM, diabetes mellitus; HT, hypertension; COPD, chronic obstructive pulmonary disease; MI, myocardial infarction; CKD, chronic kidney disease; SCr, serum creatinine; ACS, acute coronary syndrome; STEMI, ST-segment elevation myocardial infarction; NSTEMI, non-ST-elevation myocardial infarction; UA, unstable angina; LVEF, left ventricular ejection fraction; CCB, calcium channel blocker; ACE-I, angiotensin-converting enzyme inhibitor; ARB, angiotensin II receptor blocker; MACEs, major adverse cardiovascular events; event-free (A), free events from MACEs.

**Table 2:** Patients' characteristics of composite outcomes of hospitalization (ACS, HF, or stroke), hospitalization due to ACS, and hospitalization due to HF.

Characteristics	Event-free (B) (*n* = 390)		Composite hospitalization (*n* = 49)		*P* value	Event-free (C) (*n* = 391)		Hospitalization due to ACS (*n* = 34)		Hospitalization due to HF (*n* = 14)		*P* value
	*n*	%	*n*	%		*n*	%	*n*	%	*n*	%	
Age (year)												
≤65	196	50.3	23	46.9	0.762	197	50.4	17	50.0	5	35.7	0.616
>65	194	49.7	26	53.1		194	49.6	17	50.0	9	64.3	

Male	223	57.2	26	53.1	0.647	224	57.3	20	58.8	5	35.7	0.306
HT	231	59.2	34	69.4	0.215	232	59.3	23	67.7	10	71.4	0.495
DM	116	29.7	18	36.7	0.326	116	29.7	11	32.4	7	50.0	0.242
Dyslipidemia	175	44.9	31	63.3	0.022	175	44.8	20	58.8	11	78.6	0.017
COPD	11	2.8	2	4.1	0.646	12	3.1	0	0.0	1	7.1	0.286
Smoking	45	11.5	3	6.1	0.335	45	11.5	3	8.8	0	0.0	0.491
Prior MI	15	3.9	3	6.1	0.439	15	3.8	1	2.9	2	14.3	0.147
CKD	15	3.9	5	10.2	0.060	15	3.8	1	2.9	4	28.6	0.004

SCr (mg/dL)												
≤1.1	201	51.5	20	40.8	0.174	202	51.7	16	47.1	3	21.4	0.077
>1.1	189	48.5	29	59.2		189	48.3	18	52.9	11	78.6	

Type of ACS												
NSTEMI & UA	247	63.3	33	37.4	0.639	248	63.4	22	64.7	10	71.4	0.907
STEMI	143	36.7	16	32.7		143	36.6	12	35.3	4	28.6	

Reperfusion or revascularization					

No	146	37.4	18	36.7	1.000	146	37.3	9	26.5	9	64.3	0.054
Yes	244	62.6	31	63.3		245	62.7	25	73.5	5	35.7	

LVEF (%)												
>40	274	70.3	25	51.0	0.009	274	70.1	22	64.7	3	21.4	0.001
≤40	116	29.7	24	49.0		117	30.0	12	35.3	11	78.6	

Medication												
Aspirin	379	97.2	48	98.0	1.000	380	97.2	33	97.1	14	100.0	1.000
*β*-blocker	291	74.6	34	69.4	0.489	291	74.4	25	73.5	9	64.3	0.676
CCB	65	16.7	7	14.3	0.838	65	16.6	5	14.7	2	14.3	1.000
ACE-I/ARB	234	60.0	23	47.0	0.091	235	60.1	17	50.0	5	35.7	0.121
Statin	259	66.4	34	69.4	0.749	260	66.5	24	70.6	9	64.3	0.872
Influenza vaccination	205	52.6	16	32.7	0.010	206	52.7	11	32.4	4	28.6	0.020

DM, diabetes mellitus; HT, hypertension; COPD, chronic obstructive pulmonary disease; MI, myocardial infarction; CKD, chronic kidney disease; SCr, serum creatinine; HF, heart failure; ACS, acute coronary syndrome; STEMI, ST-segment elevation myocardial infarction; NSTEMI, non-ST-elevation myocardial infarction; UA, unstable angina; LVEF, left ventricular ejection fraction; CCB, calcium channel blocker; ACE-I, angiotensin-converting enzyme inhibitor; ARB, angiotensin II receptor blocker; Composite hospitalization, composite hospitalization due to ACS, HF, or stroke; event-free (B), free events from composite hospitalization due to ACS, HF, or stroke; event-free (C), free events from hospitalization due to ACS or HF.

**Table 3:** Multivariable hazard ratios stratified by influenza vaccination for each cardiovascular event, which was analyzed by multivariable stratified Cox's regression analysis.

Prognostic indicators	No vaccination		Influenza vaccination	
	HR (95% CI)	*P *value	HR (95% CI)	*P* value
MACEs				
LVEF (%)				
≤40	2.07 (1.12–3.82)	0.021	2.37 (1.01–5.59)	0.048
Medication				
ACE-I/ARB	0.44 (0.23–0.83)	0.012	1.12 (0.45–2.78)	0.806
Death				
Age (year)				
>65	10.78 (1.39–83.62)	0.023	2.28 (0.42–12.48)	0.341
Medication				
ACE-I/ARB	0.26 (0.07–0.94)	0.041	1.15 (0.21–6.30)	0.870
Composite hospitalization due to ACS, HF, or stroke				
LVEF (%)				
≤40	2.25 (1.14–4.45)	0.020	2.16 (0.81–5.76)	0.124
Medication				
ACE-I/ARB	0.48 (0.24–0.99)	0.046	1.23 (0.43–3.54)	0.701
Hospitalization due to ACS				
No indicator was found				
Hospitalization due to HF				
CKD	5.12 (1.27–20.65)	0.022	24.01 (1.38–417.20)	0.029
LVEF (%)				
≤40	7.93 (1.63–38.66)	0.010	8.37 (0.72–97.72)	0.090
Medication				
Beta-blocker	1.63 (0.34–7.78)	0.542	0.05 (0.003–0.76)	0.037

MACEs, major adverse cardiovascular events; LVEF, left ventricular ejection fraction; ACE-I, angiotensin-converting enzyme inhibitor; ARB, angiotensin II receptor blocker; ACS, acute coronary syndrome; HF, heart failure; CKD, chronic kidney disease.

**Table 4:** Discrimination of multivariable hazard ratios by the influenza vaccination for each cardiovascular event.

Prognostic indicators	No vaccination	Influenza vaccination	*Z*	*P* value
	HR (95% CI)	HR (95% CI)		
MACEs				
LVEF (%)				
≤40	2.07 (1.12–3.82)	2.37 (1.01–5.59)	−0.26	0.797
Medication				
ACE-I/ARB	0.44 (0.23–0.83)	1.12 (0.45–2.78)	−1.65	0.098
Death				
Age (year)				
>65	10.78 (1.39–83.62)	2.28 (0.42–12.48)	1.14	0.252
Medication				
ACE-I/ARB	0.26 (0.07–0.94)	1.15 (0.21–6.30)	−1.38	0.169
Composite hospitalization due to ACS, HF, or stroke				
LVEF (%)				
≤40	2.25 (1.14–4.45)	2.16 (0.81–5.76)	0.07	0.948
Medication				
ACE-I/ARB	0.48 (0.24–0.99)	1.23 (0.43–3.54)	−1.43	0.152
Hospitalization due to ACS				
No indicator was found				
Hospitalization due to HF				
CKD	5.12 (1.27–20.65)	24.01 (1.38–417.20)	−0.95	0.340
LVEF (%)				
≤40	7.93 (1.63–38.66)	8.37 (0.72–97.72)	−0.04	0.971
Medication				
Beta-blocker	1.63 (0.34–7.78)	0.05 (0.003–0.76)	2.18	0.030

MACEs, major adverse cardiovascular events; LVEF, left ventricular ejection fraction; ACE-I, angiotensin-converting enzyme inhibitor; ARB, angiotensin II receptor blocker; ACS, acute coronary syndrome; HF, heart failure; CKD, chronic kidney disease.

**Table 5:** Multivariable hazard ratios and 95% confidence intervals of influenza vaccination and beta-blocker for hospitalization due to HF.

Influenza vaccine	Beta-blocker	HR	95% CI	*P* value
No	No	Reference		
No	Yes	1.29	0.27–6.16	0.750
Yes	No	2.46	0.40–15.22	0.334
Yes	Yes	0.05	0.01–0.71	0.027

*Note*. All analyses were adjusted for gender, dyslipidemia, SCr, and LVEF, which are independent prognostic indicators for hospitalization due to HF.
